# Outcomes of allogeneic transplantation in blast-phase myeloproliferative neoplasms: one-third achieve long-term survival

**DOI:** 10.1038/s41409-026-02842-z

**Published:** 2026-04-16

**Authors:** Kordelia Barbullushi, Nico Gagelmann, Kristin Rathje, Anita Badbaran, Johanna Richter, Mathias Schäfersküpper, Franziska E. Marquard, Radwan Massoud, Evgeny Klyuchnikov, Normann Steiner, Mirjam Reichard, Ina Rudolph, Silke Heidenreich, Christian Niederwieser, Catherina Lück, Dietlinde Janson, Christine Wolschke, Francis Ayuk, Nicolaus Kröger

**Affiliations:** 1 Hematology Unit, Foundation IRCCS Ca’ Granda Ospedale Maggiore Policlinico, Milan, Italy; 2 Department of Stem Cell Transplantation, University Medical Center Hamburg-Eppendorf, Hamburg, Germany

**Keywords:** Myeloproliferative disease, Acute myeloid leukaemia

## Abstract

Philadelphia-negative myeloproliferative neoplasms (MPNs) can progress to blast phase (MPN-BP), a biologically distinct and highly lethal entity with a median survival typically under six months. Allogeneic hematopoietic stem cell transplantation (allo-HSCT) remains the only potentially curative approach, yet relapse and non-relapse mortality limit durable benefit. We retrospectively analyzed post-transplant outcomes in 51 consecutive adults undergoing 53 allo-HSCTs for MPN-BP. Median age was 62 years; most cases evolved from myelofibrosis, and JAK2 was the predominant driver mutation. Neutrophil engraftment occurred in all but two patients (median 12 days). At 1-year, cumulative incidences were 35.8% for grade II-IV acute GVHD, 7.5% for moderate-severe chronic GVHD, 44.3% for relapse, and 25.8% for non-relapse mortality. One-year overall survival (OS) and disease-free survival were 43.4% and 37.7%, respectively; relapse was the leading cause of death. In multivariable analysis, *TP53* mutations and higher peripheral blast burden adversely affected OS, while *CALR* mutations appeared to be associated with improved OS, peripheral blasts also independently predicted relapse. These data underscore the cure rate of approximately one-third of MPN-BP and highlight peripheral blasts and *TP53* as actionable risk markers for transplant strategies.

## Introduction

The Philadelphia-negative myeloproliferative neoplasms (MPNs) are a group of clonally related disorders, including polycythemia vera (PV), essential thrombocythemia (ET), and primary or secondary myelofibrosis (PMF and SMF, respectively). These disorders are characterized by constitutive activation of the JAK2 signaling pathway, which in almost 90% of cases is secondary to usually mutually exclusive mutations in the driver genes (*JAK2, CALR, MPL*) [1]. In the natural history of these diseases, the allele burden of driver mutations may increase, and additional mutations may be acquired in genes encoding epigenetic regulators, transcription factors (especially *TP53*), and splicing factors [2]. This, along with the development of chromosomal aberrations and persistent cytokine-driven inflammation, can lead to clonal progression through an accelerated phase (AP) up to an overt leukemic transformation [3]. The latter is defined blast-phase (BP), for which diagnostic criteria require a blast count of ≥20% in the bone marrow and/or in the peripheral blood [4]. The risk of evolution to BP at 20 years has been reported at 4-7% for ET and PV, and 14% for PMF [4, 5]. A specific inner risk of leukemic transformation seems to be associated with different driver mutation status: 19.4% in *JAK2*-mutant, 9.4% in *CALR*-mutant, 16.9% in *MPL*-mutant and 34.4% in triple-negative patients [6, 7].

The prognosis of this leukemic transformation is poor, with median overall survival (OS) reported to be under six months. MPN-BP emerges as an aggressive disease marked by clonal, functional, and phenotypic heterogeneity, resulting in a biologically distinct entity compared to de novo acute myeloid leukemia (AML). Currently available treatments, including both intensive (chemotherapy) and non-intensive treatments (hypomethylating agents [HMA] alone or in combination with BCL-2 inhibitor venetoclax and/or targeted therapies), have shown limited and short-lasting efficacy and do not significantly improve long-term survival [7–9]. At present, allogeneic hematopoietic stem cell transplantation (allo-HSCT) is the only treatment capable of offering curative potential and sustained long-term survival [10–13]. The main limitations of this strategy include high rates of relapse and transplant-related mortality, together with its restricted applicability to a limited subset of eligible patients.

In this study, we described the post-transplant outcomes of a retrospective cohort of 51 consecutive patients receiving allo-HSCT with curative intent for MPN-BP.

## Methods

### Study design

This single-center retrospective cohort study assessed post-transplant outcomes after allogeneic hematopoietic stem cell transplantation (allo-HSCT) for myeloproliferative neoplasm in blast phase (MPN-BP) at the University Medical Center Hamburg-Eppendorf (Hamburg, Germany). Adult patients (≥18 years) who underwent allo-HSCT for MPN-BP through 2020 were included. All consecutive transplants were eligible irrespective of stem cell source, conditioning regimen, or graft-versus-host disease (GVHD) prophylaxis [14, 15]. Donor categories were matched related donor (MRD), matched unrelated donor (MUD), mismatched unrelated donor (MMUD), and haploidentical (haplo). Remission status at transplantation was assigned according to current international criteria [16]. When available, molecular and cytogenetic data were classified using MIPSS70+ version 2.0 [17]. All patients provided informed consent in accordance with the Declaration of Helsinki guidelines.

### Endpoints

The primary endpoint was OS (defined as time from allo-HSCT to death from any cause). Secondary endpoints were disease-free survival (DFS, defined as time from allo-HSCT to relapse/progression or death, whichever occurred first), relapse incidence (RI, recurrence of the underlying hematologic malignancy after allo-HSCT), non-relapse mortality (NRM, as death without prior relapse/progression), and cumulative incidence of neutrophil engraftment and acute or chronic GVHD (aGVHD, cGVHD, defined per established consensus criteria) [18, 19].

The probabilities of DFS and OS were estimated using the Kaplan-Meier method, with univariate survival comparisons conducted using the log-rank test. Rates of aGVHD, cGVHD, RI and NRM were calculated using the cumulative incidence estimator to account for competing risks. In the case of NRM, relapse was considered the competing event, while for RI, the competing risk was death without relapse. For studying aGVHD and cGVHD, both relapse and death were considered competing events. A forest plot was generated based on a Cox proportional hazards regression analysis to evaluate the clinical variables influencing outcomes.

## Results

### Cohort description

We included 51 patients, two of whom underwent two transplants in blast phase, for a total of 53 allo-HSCTs (Table 1). Thirty-nine patients (73.6%) were at first transplant; all previously transplanted patients had received their prior allo-HSCT in chronic phase, except three with a prior blast-phase transplant (one performed at another center). The median transplant year was 2016 (range 2002–2020), and the median age was 62 years. The most common indication was BP arising from PMF (56.8%), followed by BP from post-PV SMF (19.6%), PV (11.8%), post-ET SMF (9.8%), and ET (2%). Driver mutations were JAK2 in 56.8%, CALR in 19.6%, MPL in 2%, and triple-negative in 9.8%. High-risk mutations per MIPSS70 + v2.0 were assessable in 38 patients (71.6%), of whom 36.8% harbored at least one; TP53 status was available in 32 patients, with mutations identified in three.

**Table 1:** Patient-, disease- and transplant-characteristics of the study population.

Year of transplantation, median (range)	2016 (2002–2020)
Transplantation, *n* (%)	
First	39 (73.6)
Second	13 (24.5)
Third	1 (1.9)
Patient age, median (range)	62 (43–75)
Patient sex, *n* (%)	
Male	29 (56.9)
Female	22 (43.1)
Diagnosis, *n* (%)	
BP PMF	29 (56.8)
BP SMF post-ET	5 (9.8)
BP SMF post-PV	10 (19.6)
BP post-ET	1 (2)
BP post-PV	6 (11.8)
Driver mutation status, *n* (%)	
*JAK2*	30 (58.8)
*CALR*	10 (19.6)
*MPL*	1 (2)
Triple negative	5 (9.8)
Missing	5 (9.8)
High risk mutation according to MIPSS-70^+^ version 2.0, *n* (%)	
Yes	14 (27.5)
No	24 (47)
Missing	13 (25.5)
Cytogenetics according to MIPSS-70^+^ version 2.0, *n* (%)	
Standard risk	19 (37.3)
Unfavourable	5 (9.8)
Very high risk	22 (43.1)
Missing	5 (9.8)
*TP53* status, *n* (%)	
Mutated	3 (5.9)
Wild type	29 (56.9)
Missing	19 (37.2)
% of blasts in pre-treated patients, median (range)	3 (0-81)
% of blasts in not pre-treated patients, median (range)	25 (20–61)
% of blasts in patients receiving sequential conditioning, median (range)	20 (0–81)
% of blasts in patients receiving standard conditioning, median (range)	2 (0–58)
Splenectomy prior to transplant, *n* (%)	5 (9.8)
Spleen irradiation prior to transplant, *n* (%)	1 (1.9)
Donor type, *n* (%)	
MRD	7 (13.2)
MUD	31 (58.5)
MMUD	13 (24.5)
Haploidentical donor	2 (3.8)
CD34+ cell dose x 10^6^/kg, median (range)	7.3 (2.4–11.4)
Stem cell source, *n* (%)	
PBSC	52 (98.1)
BM	1 (1.9)
Donor age, median (range)	39 (18–64)
Female donor to male recipient, *n* (%)	5 (9.4)
Compatibility AB0, *n* (%)	
Match	19 (35.8)
Minor mismatch	10 (18.9)
Major mismatch	16 (30.2)
Bidirectional mismatch	8 (15.1)
CMV serology status patient/donor, *n* (%)	
–/–	12 (22.6)
+/+	30 (56.6)
–/+	2 (3.8)
+/–	9 (17)
Conditioning intensity, *n* (%)	
RIC	44 (83)
MAC	9 (17)
Conditioning, *n* (%)	
TreoFlu	8 (15.1)
BuFlu	10 (18.9)
FluTBI	2 (3.8)
TTBu	2 (3.8)
FLAMSA-BuFlu	17 (32.1)
FLAMSA-Treo	4 (7.5)
FLAMSA-CyTBI	3 (5.7)
FLAMSA-Bu	2 (3.8)
Other	5 (9.4)
T-cell depletion, *n* (%)	
ATLG-Neovii	38 (71.6)
ATG-Thymoglobulin	8 (15.1)
PTCy	3 (5.7)
No TCD	2 (3.8)
Missing	2 (3.8)
GVHD Prophylaxis, *n* (%)	
CSA-MMF	42 (79)
CSA-MTX	6 (11)
Tacrolimus-MMF	5 (9)

*BP* blast phase, *PMF* primary myelofibrosis, *SMF* secondary myelofibrosis, *ET* essential thrombocythemia, *PV* polycythemia vera, *MRD* matched related donor, *MUD* matched unrelated donor, *MMUD* mismatched unrelated donor, *PBSC* peripheral blood stem cell, *BM* bone marrow, *CMV* cytomegalovirus, *MAC* myeloablative conditioning, *RIC* reduced intensity conditioning, *Treo* treosulfan, *Flu* fludarabine, *Bu* busulfan, *TBI t*otal body irradiation, *TT* thiotepa, *FLAMSA* fludarabine-amsacrine-cytarabine, *Cy* cyclophosphamide, *ATLG* Anti-T-Lymphocyte Globulin, *ATG* Anti-Thymocyte Globulin, *PTCy* post-transplant cyclophosphamide, *TCD* T-cell depletion, *GVHD* graft-versus-host disease, *CSA* cyclosporine, *MMF* mycophenolate mofetil, *MTX* methotrexate, *n* number.

Overall, 34 patients (64.2%) received induction therapy: 31 (58.5%) intensive chemotherapy, one azacitidine alone, and two sequential chemotherapy and azacitidine. Five patients previously transplanted in the chronic phase received DLI, with chemotherapy added after DLI failure in three cases. Among pretreated patients, median blasts at transplant was 3%, and 47% achieved CR/CRi (ALR-P 32%, ALR-C 15%). Nineteen patients (35.8%) proceeded directly to allo-HSCT without prior therapy, with a median blast burden of 25% at transplant.

31 patients (58.5%) received the graft from a MUD, 13 (24.5%) from a MMUD, 7 (13.2%) from a MRD and 2 (3.8%) from a haploidentical donor. Peripheral blood was the source of stem cells used in 52 patients (98.1%). Almost half of the population (28 patients, 52.8%) received a FLAMSA-based sequential conditioning: 14 of them after an induction treatment, clearing blasts at < 5% in 6 cases; the remaining 22 patients underwent sequential conditioning while still in active leukemia. Overall, patients receiving sequential conditioning had a median blast percentage of 20%, while those receiving standard conditioning had a median blast percentage of 2%. Among patients undergoing standard conditioning, the majority were treated with either busulfan-fludarabine (10 patients, 18.9%) or treosulfan-fludarabine (8 patients, 15.1%). Overall, most of the patients, including those with sequential conditioning, received an RIC. As for GVHD prophylaxis, all patients received a backbone of calcineurin inhibitor-antimetabolite (CSA-MMF in 79%), with further addition of in vivo T-cell depletion in 49 patients (92.4%). Post-transplant cyclophosphamide (PTCy) was used in haploidentical transplants and in 1 case of MUD-transplant. ATLG-Neovii was more frequently used compared to ATG-Thymoglobulin.

### Post-transplant outcomes

**Fig. 1:**
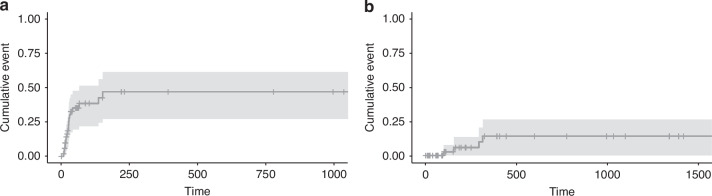
Cumulative incidence of graft-versus-host disease in the study cohort. **a** Grade II–IV acute GVHD. **b** Moderate-to-severe chronic GVHD.

**Fig. 2:**
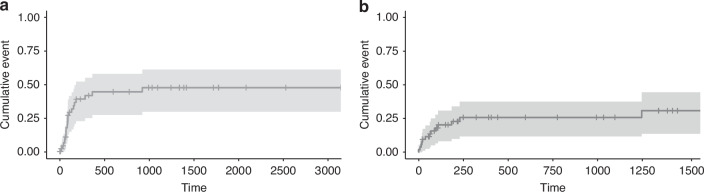
Cumulative incidence of relapse and non-relapse mortality in the study cohort. **a** RI. **b** NRM.

All patients except two engrafted with a median time to neutrophil engraftment of 12 days (range 6–24). Among patients missing engraftment, one experienced primary graft failure, and the other one died because of an infectious complication during conditioning. 4 patients developed poor graft function, and 1 patient developed secondary graft failure. At 1 year, cumulative incidence of grade II-IV aGVHD was 35.8% and of moderate-severe cGVHD was 7.5% (Fig. 1). At 1 year, cumulative incidence of RI was 44.3% (95% CI 27–57.5%) and NRM was 25.8% (95% CI 11.8–37.7) (Fig. 2).

**Fig. 3:**
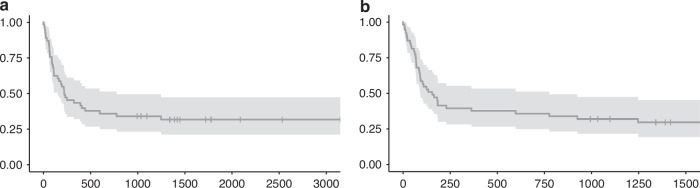
Overall survival and disease-free survival in the study cohort. **a** OS **b** DFS.

The median follow-up time for surviving patients was 4.7 years. The 1-year OS was 43.4% (95% CI 31.9–59) and the 1-year DFS was 37.7% (95% CI 11.8–37.7; Fig. 3). The 5-year OS according to type HSCT was 35.95% (95% CI, 22.58–57.24%) for patients undergoing first HSCT and 28.57% (95% CI, 12.48–65.41%) for those after second HSCT. The primary cause of death was relapse/progression of the disease (16 patients), followed by infectious complications (9 patients), GVHD (3 patients), other transplant-related complications (4 patients); in addition, for 2 patients, the cause of death has not been collected.

### Potential factors on outcomes

**Fig. 4:**
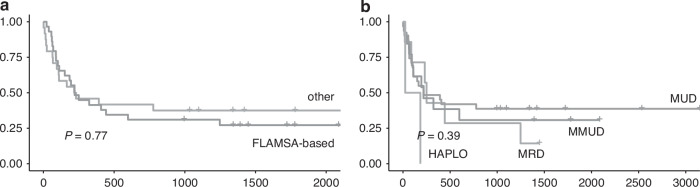
Overall survival according to transplant-related variables. **a** OS in FLAMSA-based conditioning vs other conditioning **b** OS in MRD vs MUD vs MMUD vs haplo.

First, we applied subgroup analysis for conditioning regimen and donor type (Fig. 4). We observed that no significant differences were reported between FLAMSA-based conditioning and standard conditioning, while MUD was associated with a tendency towards higher OS, although not statistically significant. The only two patients receiving the graft from a haploidentical donor died from infectious complications.

Second, a subgroup analysis of OS was performed for ELN 2022 molecular categories. Among 47 evaluable patients, 21 were classified as intermediate-risk and 26 as unfavorable-risk. Median OS was numerically longer in the intermediate-risk group (1.2 years) compared with the unfavorable-risk group (0.3 years), but this difference did not reach statistical significance (log-rank *p* = 0.24). These findings indicate that ELN 2022 stratification underlines dismal prognosis, with prognostic discrimination for OS following allo-HSCT in MPN-BP, likely due to disease biology convergence in blast phase and limited statistical power.

Third, to explore potential era effects, the transplant year was evaluated as a continuous variable in a Cox model. No significant association between transplant date and OS was observed (HR 1.00, *p* = 0.17), although the limited sample size and number of events constrain the statistical power of this analysis.

**Fig. 5:**
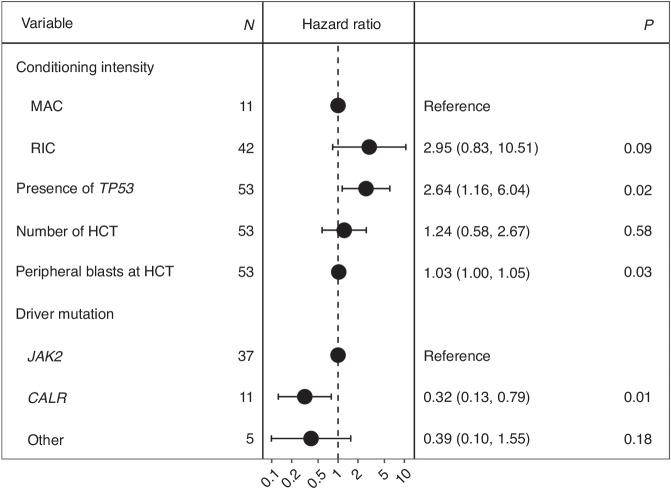
Multivariate analysis of overall survival in the study cohort. Forest plot of hazard ratios for disease- and transplant-related variables.

**Fig. 6:**
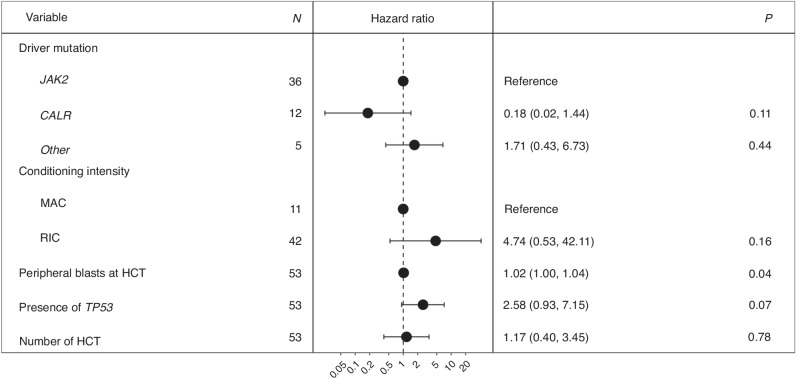
Multivariate analysis of relapse incidence in the study cohort. Forest plot of hazard ratios for disease- and transplant-related variables.

Next, we applied multivariable modeling to adjust for potential risk factors and evaluate independent effects of genetic, disease-, and transplant-related features on OS and RI (Fig. 5 and Fig. 6). Our analysis revealed that variables negatively impacting OS included the presence of *TP53* mutations (HR 2.64 [95% CI 1.16–6.04], *p* = 0.02) and the rate of peripheral blasts (HR 1.03 [95% CI 1.00–1.05], *p* = 0.03), with each 1% increase in circulating blasts at time of HSCT corresponding to a 3% increase in the risk of death. In contrast, the presence of *CALR* mutations was associated with improved OS compared to other driver mutations (HR 0.32 [95% CI 0.13–0.79], *p* = 0.01). No other variables in our cohort had a significant impact on OS. Peripheral blasts also independently predicted a higher risk of RI in multivariate analysis (HR 1.02 [95% CI 1.00–1.04], *p* = 0.04).

## Discussion

In this single-center cohort of patients with blast-phase MPN undergoing allo-HSCT, approximately one-third achieved durable long-term survival, confirming transplantation as the only potentially curative option despite high early relapse and NRM. Multivariable analysis indicated that post-transplant outcomes were primarily driven by disease-related factors, with *TP53* and higher peripheral blast burden at transplant independently associated with inferior survival. Conditioning intensity did not reach statistical significance in the multivariable model; however, RIC was associated with a numerically higher hazard compared with MAC. Driver mutation status retained prognostic relevance, while other transplant-related variables had no independent impact on survival.

Allo-HSCT stands out as the sole potentially curative option for blast-phase MPN, with worse outcomes compared to de novo chronic MPN or post-myelodysplastic neoplasm AML, and updated EBMT guidelines now address transplant management in this setting [10]. Disease-related scores play a crucial role in determining potential transplantation indications for fit patients. These decisions are based on the expected natural history of the disease, which is assessed using various criteria: clinical features, molecular and cytogenetic characteristics, or a combination of these parameters. For the specific setting of AP myelofibrosis, a retrospective study showed great long-term survival for patients proceeding to transplant when still in AP [20]. When eligible, patients in this category should be promptly referred for allo-HSCT before overt transformation. Moreover, the evaluation of transplant-specific prognostication is recommended by the EBMT guidelines to identify patients who may present an intrinsic transplant-related probability of lower OS, while it was implemented in chronic phase myelofibrosis [21].

In transplant-eligible patients, induction therapy—using intensive chemotherapy or HMA-based regimens with or without venetoclax or targeted agents—is commonly pursued before transplantation, but no optimal strategy is established, as evidence is largely retrospective or derived from single-arm studies. No current strategy has demonstrated clear superior efficacy, with comparable overall response rates around 40–50% and no long-term survival benefits in the absence of allo-HSCT [22–24]. Recently, a retrospective multicenter analysis conducted on 202 patients confirmed the lack of superiority among currently available front-line treatment approaches (chemotherapy vs. HMA alone vs. HMA + venetoclax) [23]. Further therapeutic options may include the use of JAK inhibitors, widely used in chronic phase myelofibrosis, in combination with HMA, or CPX-351 [25, 26]. However, available small-sized reports have failed to show any advantage in terms of response. In our study, most of the patients received an intensive induction through chemotherapy. Only 3 patients received single-agent azacytidine: 2 failed induction and proceeded directly to transplant, and 1 achieved CR with second-line chemotherapy after failure of HMA. Considerations about the role of induction treatment and remission status prior to transplant rely mainly on retrospective series, showing contradictory results [11–13, 27]. An alternative approach for managing blast burden may involve the use of sequential conditioning, originally developed for high-risk AML, particularly primary refractory and relapsed cases [28]. Sequential conditioning combines short-course intensive chemotherapy (e.g., FLAMSA) with subsequent, usually reduced-intensity, transplant conditioning to lower leukemia burden [29]. Although nearly half the cohort—mainly with active disease—received FLAMSA-based conditioning, no OS benefit was observed, likely confounded by higher baseline disease burden; conditioning intensity likewise showed no significant association with OS, consistent with prior reports. Relapse is the main reason for treatment failure, with a cumulative incidence of 44% at one year in our study, which was slightly lower than previously reported. Relapse mechanisms may arise from intrinsic evolution of the disease, with modifications in its mutational landscape, or from immune evasion strategies [30–34]. Relapse management includes anti-leukemic therapies (intensive or non-intensive reinduction and targeted agents when available), DLI, interferon, and selected second transplants; DLIs are effective in post-transplant relapse of chronic-phase myelofibrosis, though data in MPN-BP are limited. Detection of driver mutations is a known predictor of long-term transplant outcomes in myelofibrosis, but leukemia can develop from clones that lack driver mutations, indicating that other markers for relapse evolution are needed [34–36]. While pre-transplant CR was not associated with improved OS, higher blast burden correlated with increased mortality, underscoring the prognostic relevance of disease burden at transplant, consistent with observations in primary refractory de novo AML transplanted with active disease. However, defining the optimal induction treatment for this setting remains an unresolved challenge. Previous studies identified genetic features, rather than remission status, as the primary predictors of OS in MPN-BP patients [12, 35]. We confirmed a significant association between *TP53* mutations and reduced OS. *TP53* is a well-established prognostic factor in myeloid neoplasms, now strongly confirmed also for both chronic-phase and BP-MPN [37]. However, driver mutations can also influence long-term outcomes [30]. We found that *CALR* mutations were associated with longer OS compared to *JAK2*, *MPL*, or triple-negative cases, although power was small and further investigation is needed. Key limitations include the retrospective, single-center design and the long study period (2002-2020), during which induction strategies, conditioning/GVHD prophylaxis, and supportive care evolved, introducing temporal confounding. The cohort is small, limiting power and yielding imprecise estimates, particularly in clinically important subgroups (e.g., TP53-mutated disease, haploidentical donors, second transplants), and increasing the risk of false-negative findings. Molecular annotation and response assessment were incomplete and non-uniform (notably missing TP53 data in many cases and no standardized MRD strategy), which may limit conclusions about the role of cytoreduction versus residual disease. Finally, marked clinical heterogeneity and confounding by indication—especially higher blast burden among patients receiving FLAMSA-based sequential conditioning—restrict causal inference and generalizability beyond this center’s selection and practice patterns. Overall, allo-HSCT is confirmed as the only option able to rescue almost one third of the patients, prolonging long-term disease-free survival in a context in which, otherwise, no possibility of cure would be feasible with available therapies.

## Data Availability

Data can be requested by email to the corresponding author.
